# DNA-Stable Isotope Probing Shotgun Metagenomics Reveals the Resilience of Active Microbial Communities to Biochar Amendment in Oxisol Soil

**DOI:** 10.3389/fmicb.2020.587972

**Published:** 2020-11-17

**Authors:** Julian Yu, Michael J. Pavia, Lauren M. Deem, Susan E. Crow, Jonathan L. Deenik, Christopher Ryan Penton

**Affiliations:** ^1^School of Life Sciences, Arizona State University, Tempe, AZ, United States; ^2^Center for Fundamental and Applied Microbiomics, The Biodesign Institute, Arizona State University, Tempe, AZ, United States; ^3^Swette Center for Environmental Biotechnology, The Biodesign Institute, Arizona State University, Tempe, AZ, United States; ^4^Natural Resources and Environmental Management, University of Hawai‘i at Mânoa, Honolulu, HI, United States; ^5^Tropical Plant and Soil Sciences, University of Hawai‘i at Mânoa, Honolulu, HI, United States; ^6^College of Integrative Sciences and Arts, Arizona State University, Mesa, AZ, United States

**Keywords:** biochar amendment, isopycnic centrifugation, carbon sequestration, denitrification, Nextseq sequencing, metagenomic assembled genomes, active bacterial populations

## Abstract

The functions and interactions of individual microbial populations and their genes in agricultural soils amended with biochar remain elusive but are crucial for a deeper understanding of nutrient cycling and carbon (C) sequestration. In this study, we coupled DNA stable isotope probing (SIP) with shotgun metagenomics in order to target the active community in microcosms which contained soil collected from biochar-amended and control plots under napiergrass cultivation. Our analyses revealed that the active community was composed of high-abundant and low-abundant populations, including *Actinobacteria, Proteobacteria, Gemmatimonadetes*, and *Acidobacteria*. Although biochar did not significantly shift the active taxonomic and functional communities, we found that the *narG* (nitrate reductase) gene was significantly more abundant in the control metagenomes. Interestingly, putative denitrifier genomes generally encoded one gene or a partial denitrification pathway, suggesting denitrification is typically carried out by an assembly of different populations within this Oxisol soil. Altogether, these findings indicate that the impact of biochar on the active soil microbial community are transient in nature. As such, the addition of biochar to soils appears to be a promising strategy for the long-term C sequestration in agricultural soils, does not impart lasting effects on the microbial functional community, and thus mitigates un-intended microbial community shifts that may lead to fertilizer loss through increased N cycling.

## Introduction

Modern agriculture faces multiple challenges: it must produce more food and fiber to feed a growing global population, adopt more efficient and sustainable management strategies for production, and adapt to climate change (FAO, 2017). These challenges require action by restructuring agroecosystems in order to increase food production from existing farm land while concomitantly achieving major reductions in environmental impacts (Garnett et al., 2013; Campbell et al., 2014). In this regard, incorporation of biochar into soils is a promising management strategy to address the reduction of greenhouse gas (GHG) emissions by enhancing carbon (C) sequestration in agricultural soils, while concurrently improving soil fertility (Jha et al., 2010; Lehmann et al., 2011; Paustian et al., 2016). Biochar is a carbonaceous product of biomass pyrolysis, which contains large portions of aromatic compounds that influence its stability and the spatial organization of C within soil particles (Wiedemeier et al., 2015; Hernandez-Soriano et al., 2016). In addition to carbon sequestration, biochar is intended for use as a soil conditioner to improve soil properties relevant to crop productivity (Jeffery et al., 2011). The hypothesized mechanisms for positive improvements to soil fertility are often explained by increased cation exchange capacity (CEC) (Liang et al., 2006), porosity, liming capacity, as well as enhanced water and nutrient retention (Lehmann et al., 2011; Laghari et al., 2016), and its influence on soil structure (Jien and Wang, 2013). However, the utility of biochar for any particular application is dependent on the soils it is added to and the biochars inherent properties, which are a function of feedstock and pyrolysis temperature. Previous studies have shown that feedstock type largely determines the CEC, mineral elements concentration and total organic carbon (OC) (Mukherjee et al., 2011; Barrow, 2012; Zhao et al., 2013). Enriched biochar can also be made by mixing biochars with minerals, clays and manure and heating the mixture at low temperatures to increase the concentration of exchangeable cations and available phosphorus (Chia et al., 2014). While parameters that most affect pH, surface chemistry, recalcitrance, and volatile matter content in biochar are mainly influenced by pyrolysis temperature, with recalcitrance increasing and volatile matter content decreasing with higher pyrolysis temperatures (Bruun et al., 2011; Zhao et al., 2013; Suliman et al., 2016). The ability to tailor biochar through feedstock and pyrolysis manipulations offers considerable opportunities for the use of biochar as a soil conditioner or carbon negative technology.

Despite the widespread interest in the application of biochar as a sustainable management practice, the effects of biochar on the soil microbiome still remain relatively underexplored due to the vast metabolic and phylogenetic diversity of the microorganisms present in soils. Soil microbial communities are complex and play key roles in sustaining soil function due to their significant role in regulating global nutrient and biogeochemical cycling via fundamental ecological processes such as mineralization and decomposition. The addition of biochar to soils can modify the soil environment which can influence soil microbial activity, abundance, and community composition. However, the microbial response to biochar is dependent on numerous factors such as soil type, properties of the biochar and cropping system (Anders et al., 2013; Docherty et al., 2015; Jenkins et al., 2017; Yu et al., 2018). In short-term experiments lasting less than 1 year, biochar produced at lower pyrolysis temperatures have been reported to increase soil respiration (Luo et al., 2011; Wang et al., 2012), while longer-term experiments (up to 4 years) have reported no significant difference in soil respiration but significantly increased soil microbial biomass (Zhang et al., 2014; Zheng et al., 2016). Other short-term studies have reported changes in the soil microbial community after biochar amendment, with increased microbial biomass after the application of biochars produced at lower pyrolysis temperatures (Anders et al., 2013; Chen et al., 2016), while others have found no significant change or decreased microbial biomass with the addition of biochar produced at higher temperatures (Elzobair et al., 2016; Li et al., 2018). Previous studies have indicated that biochar significantly altered microbial community composition, however, biochar effects have been reported with some contradictory findings on the significant changes in the relative abundance of bacterial groups. Several studies focused on shifts in the bacterial community have observed increased soil pH, water holding capacity, and nitrogen (N) mineralization and increases in the relative abundance of *Actinobacteria, Bacteroidetes*, and *Proteobacteria* (Anderson et al., 2011; Xu et al., 2016; Zheng et al., 2016). In pot-experiments examining root-associated microbial communities, biochar addition increased the relative abundance of *Bacteroidetes* and decreased *Proteobacteria* (Kolton et al., 2011). In soils that contained natural or added biochar increase soil respiration and increased relative abundance of *Gemmatimonadetes* and *Actinobacteria* have also been reported (Khodadad et al., 2011). In part, the difference observed between biochar studies may reflect the differences in soils, plant cover and the land management. Jenkins et al. (2017) examined the effects of biochar application across three site in Europe under different land management and observed that biochar increased relative abundance of *Gemmatimonadetes* and *Proteobacteria* and decreased *Acidobacteria* under an Italian grassland, while *Acidobacteria* and *Gemmatimonadetes* relative abundance increased under short rotation coppice in the United Kingdom. Other studies have found distinct bacterial communities on the surfaces of biochar or mineral enriched biochar which can support chemolithotrophic processes (Ye et al., 2017). Overall, various changes in the microbial community have been reported after biochar application though these effects are not uniform and depend strongly on soil type, biochar feedstock, application rate and cropping system (Steinbeiss et al., 2009; Khodadad et al., 2011; Lehmann et al., 2011; Gomez et al., 2014; Thies et al., 2015; Yu et al., 2018; Zhang et al., 2019). It is crucially important to understand the effects of biochar on soil microbial communities in order to predict the potential for C sequestration and nutrient cycling under large-scale agricultural production.

The ecology of soil microbial communities and changes in these communities due to biochar addition has principally been investigated using molecular techniques. These studies have primarily focused on the composition and diversity of the total community derived from soil genomic DNA, which may or may not be active. Examination of active microbial populations can provide insights into how communities respond to changing environmental conditions and contribute to nutrient cycling, C stabilization and storage. In order to predict the impact of the microbiome on soil ecosystem function, it is critical to specifically target members of the active soil microbial community. To this end, DNA stable isotope probing (SIP) is a cultivation-independent technique that can be used to link microbial activity to the identity within environmental samples (Chen and Murrell, 2010; Lee et al., 2011; Verastegui et al., 2014; Coyotzi et al., 2016). It relies on the incorporation of stable isotope labels into microbial DNA during growth on the labeled substrate, thus acting as a filter to enrich the DNA of active populations. DNA-SIP has been coupled with shotgun metagenomic sequencing to identify new functional and adaptive traits of microbial taxa and to directly link microbial populations with ecological processes (Eyice et al., 2015; Ziels et al., 2018). Metagenomic techniques have been used to study these highly complex and diverse ecosystems, providing descriptions of the taxonomic and functional potential of natural microbial communities. Additionally, assembling and binning of sequences from metagenomes has allowed for the recovery of genomes of abundant and rare microbial populations (i.e., metagenomic assembled genomes or MAGs) from various environments. However, there have been no previously reported studies coupling DNA-SIP with shotgun metagenomics to recover population MAGs from biochar-amended soils.

In the present study, we performed DNA-SIP coupled with shotgun metagenomics to investigate the active populations of the soil microbiome of a tropical Oxisol that experienced 2 years of a low-volatile matter biochar amendment under napiergrass cultivation. The objectives of this study were: (1) to identify and explore the effects of biochar amendment on active soil microbial populations, and (2) to gain insight into the functional aspects of the active community. Targeting the active community allowed for the recovery of higher quality MAGs, which enabled the characterization of gene content to test the conclusions concerning individual capabilities and metabolisms. We hypothesized that the impact of biochar on the soil microbial communities would still be apparent after 2 years following a single application of biochar. Furthermore, we hypothesized that soil microbial communities would exhibit a significant change, due to biochar addition, in the composition and abundance of the metabolically active populations and their functional responses in terms of both their nutrient cycling potential and their use of biochar-derived recalcitrant (e.g., aromatic) carbon substrates in these Oxisol soils under Napiergrass cultivation. We expected that, based on the previous metagenomic analyses (Yu et al., 2019), that biochar amendment would increase the relative abundances of two bacterial phyla in the active population, *Bacteroidetes* and *Proteobacteria*. We also expected that the active population of biochar-amended soils would exhibit an increased genetic potential for denitrification.

## Materials and Methods

### Overview of Sites, Biochar and Sample Collection

The Oxisol soils used in this study were collected from a field experiment on the island of Oahu, HI, United States at the Poamoho agricultural research station, which was managed by the College of Tropical Agriculture and Human Resources, University of Hawaii Manoa (21°32′30″N, 158°01′15″W). The soil is highly weathered with low CEC, slightly acidic, and contains about 44% clay rich in kaolinite and iron oxides (NRCS Web Soil Survey). Detailed descriptions of the field experiment, biochar type and biochar application rate were described in a previous study (Yu et al., 2018). Briefly, the biochar used in this experiment was produced by Diacarbon Inc^1^. The feedstock was 20% anaerobic digester sewage sludge and 80% spruce, pine and fir wood chips, which underwent pyrolysis in a continuous flow reactor at about 600°C. The biochar is 74% carbon, 1.03% nitrogen, and 1.93% hydrogen and has a pH of 10.53 and an electrical conductivity of 444.5 μS/cm. For proximate analysis, the biochar is 29.73% volatile matter, 56.72% fixed C and 15.31% ash. Samples were collected from plots under napiergrass (*Pennisetum purpureum* var. green bana) cultivation approximately 2 years after a single addition of biochar. Ratoon harvest was the harvest technique used for the crop, which is a zero-tillage system that retains the below-ground environment. Soil samples were collected on November 2015 from four replicate plots from biochar-amended and control soils prior to harvest. Each plot was split in half and from each half-plot three 0 – 10 cm depth cores were taken randomly and then mixed to create a composite. Four composite samples were taken per half plot for a total of 8 replicates per plot. Samples transported on dry ice to the laboratory and were frozen at −80°C without addition of any protective agent until ready for further processing. Soil chemical properties were determined as previously described (Yu et al., 2018, 2019), and are summarized in Supplementary Table S1.

### Preparation of Stable Isotope Probing Soil Microcosms

The soils, previously frozen field-moist, were thawed and soil from each plot were sieved together through a 2 mm sieve, sample replicates were composited based on the respective plot from which the soils were collected. Microcosms were prepared by adding 10 g of soil in 150 ml serum bottles and pre-incubated at 4°C open to the ambient atmosphere in the dark for 7 days to allow the soils to equilibrate. Uniformly labeled (>97 atom % ^13^C) ^13^C-perennial ryegrass (*Lolium perenne* – aboveground biomass) (IsoLife, Wageningen, Netherlands) was powdered using a mortar and pestle, 0.5% (w/w) was added to soils before bottles were sealed and capped with butyl rubber septa. Each microcosm with ^13^C-labeled perennial ryegrass was paired with an identical ‘^12^C-control’ microcosm amended with the corresponding unlabeled ^12^C-perennial ryegrass (∼1.1% atom ^13^C). ^12^C-control microcosms were used to control for background presence of GC-rich DNA in higher density CsCl gradient fractions (Youngblut and Buckley, 2014). All microcosms were maintained at 23°C for 14 days in the dark. Soil respiration as a proxy for activity was measured in parallel microcosms prepared using 5 g of soil and 0.05% (w/w) ^13^C-perennial ryegrass, soil microcosms were not continuously aerated. For determination of cumulative CO_2_ and N_2_O, 200 μl of headspace from each microcosm was sampled in triplicate using a gas-tight syringe (VICI Precision Sampling, Baton Rouge, LA, United States). Headspace CO_2_ and N_2_O content was measured on a GC-ECD-FID (SRI 8610C) after microcosms were set up (day 0) and after 1, 3, 5, 7, 10, and 14 days of incubation. A standard curve was generated prior to measurement for each time point, each standard curve contained four points ranging from 250 to 5000 ppm CO_2_ for day 0 and day 1 measurements and later from 2500 to 25,000 ppm CO_2_ for remaining gas measurements. Similarly, a four-point standard curve for was generated to determine N_2_O concentration ranging from 0.5 to 25 ppm for all time points. Cumulative gas concentrations were calculated for each microcosm by summing the aggregate gas production over the 14-day incubation.

### DNA Extraction and Density-Gradient Centrifugation

Soil samples were collected for DNA extraction from ^13^C-ryegrass fed microcosms after 14 days of incubation. Soil DNA was extracted from 5 g of soil using the DNeasy PowerMax Soil kit (Qiagen Company, Hilden, Germany) as described previously (Yu et al., 2018). An initial extraction, followed by a second successive extraction, was conducted on each sample to improve DNA extract yield. A successive extraction involved adding new aliquots of bead solution, 0.5M Tris buffer (pH 9), 0.2M phosphate buffer (pH 8) were and solution C1 to the soil pellet after initial lysis, centrifugation, and removal of supernatant containing crude DNA extract. Lysis and centrifugation steps were then repeated. The DNA extracts from the initial and successive extraction was pooled and concentrated using a DNA120 SpeedVac (Thermo Savant) and was quantified using the Qubit dsDNA high-sensitivity kit (ThermoFisher Scientific, Waltham, MA, United States) using the Qubit 3.0 (ThermoFisher Scientific, Waltham, MA, United States).

DNA extracts (3 μg DNA) were subjected to density-gradient centrifugation and fractionation (Dunford and Neufeld, 2010). Briefly, DNA extracts were mixed with gradient buffer (0.1M Tris-HCl, 0.1M KCl, and 1 mM EDTA) and 7.163M CsCl solution and loaded into 4.8ml polypropylene Quick-Seal tubes (Beckman Coulter, Brea, CA, United States). Density gradient centrifugation was performed with a VTi 65.2 rotor at 55,000 rpm at 20°C for 60 h in an OptimaMax ultracentrifuge (Beckman Coulter, Brea, CA, United States) with the vacuum on, maximum acceleration, and no brake on deceleration. Gradients were displaced with mineral oil (Johnson Johnson) pumped into the top of the Optiseal tube using a syringe pump (KD Scientific), and approximately 250 μl fractions were collected dropwise from a needle in the bottom of the tube. The temperature corrected refractive index (nD-TC 20°C) of each gradient fraction was immediately measured using an AR200 digital refractormeter (Reichart, Ithaca, NY, United States), and buoyant density was calculated from the refractive index using the equation

ρ=aη-b

where ρ is the density of the CsCl (g ml^–1^), η is the measured refractive index, and *a* and *b* are coefficient values of 10.9276 and 13.593, respectively, for CsCl at 20°C (Birnie, 1978). DNA was precipitated from each fraction as described by Dunford and Neufeld (2010). The pellet was suspended in sterile TE buffer and the final concentration of DNA in each fraction was measured using the Qubit dsDNA high-sensitivity kit (ThermoFisher Scientific, Waltham, MA, United States) using the Qubit 3.0 (ThermoFisher Scientific, Waltham, MA, United States).

### Quantitative PCR

To further detect differences in buoyant density values between the ^12^C- and ^13^C-incubated microcosms, quantitative PCR was conducted on DNA from gradient fractions with buoyant densities ranging from 1.682 to 1.719 g ml^–1^. The qPCR targeted the 16S rRNA gene fragment using the 341F/797R primer pair (Nadkarni et al., 2002). qPCR was performed using the QuantStudio3 (Applied Biosystems). Each 20 μl reaction mix contained 1 μl DNA template, 500 nM of each forward and reverse primers, 7 μl PCR-grade water, 10 μl PowerUp SYBR green Master Mix (2X, Applied Biosystems). The amplification procedure for all qPCR assays consisted of an initial denaturation at 95°C for 3 min, followed by 40 cycles of denaturation at 95°C for 45 s, annealing at 60°C for 45 s, and extension at 72°C for 1min, and a final extension at 72°C for 7 min. All samples were analyzed in duplicate, no-template controls were included on each qPCR run. Plasmid standards for qPCR were prepared by cloning the 16S rRNA PCR amplicon fragment from *E. coli* K-12 into a pCR4-TOPO plasmid using the TA TOPO cloning kit (Invitrogen, Carlsbad, CA, United States). Plasmids containing the target PCR amplicon sequence were quantified by Qubit. Gene copy numbers were calculated from the measured DNA concentration and the molecular weight of the ligated plasmid containing the PCR amplicon insert. Calibration standards included 10^8^, 10^7^, 10^6^, 10^5^, 10^4^, 10^3^, 10^2^ gene copies per reaction and included in triplicate in each qPCR run. The average slope of the calibration curve was −3.4051 (97.45% PCR efficiency) and the *R*^2^ value was 0.988.

### Metagenomic Sequencing, Assembly and Binning

**FIGURE 1:**
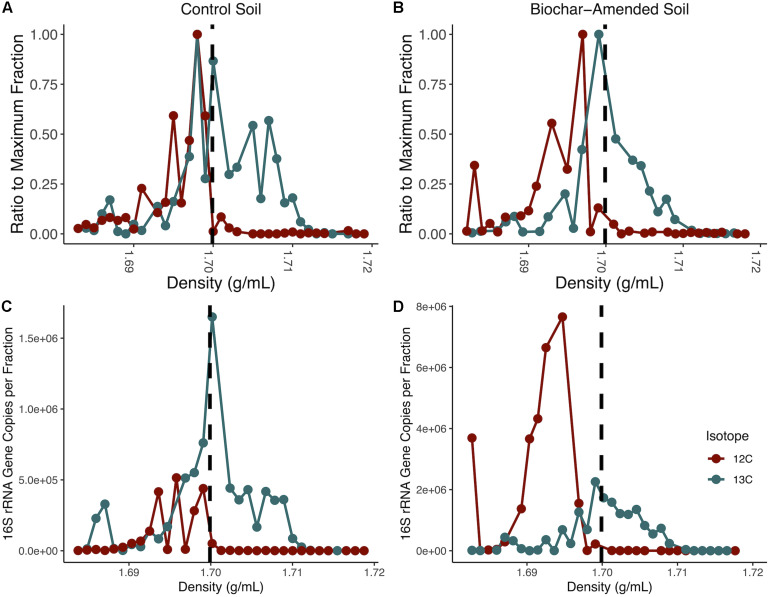
Isopycnic separation of DNA from density-gradient fractionation. Normalized DNA concentration in each fraction recovered after isopycnic separation of DNA from the ^13^C-incubated microcosms and ^12^C-controls for control soil microcosms **(A)** and biochar-amended **(B)**. DNA was measured with Qubit for each density gradient fraction, and divided by the maximum fraction value. Each point represents an average of four replicates. Gradient fractions from ^13^C-incubated microcosms were subsequently pooled for metagenomic sequencing. Total copies of 16S rRNA genes measured by qPCR for each density-gradient fraction recovered from isopycnic separation of DNA from ^13^C-incubated microcosms and ^12^C-controls for control soil microcosms **(C)** and biochar-amended microcosms **(D)**. Density gradient fractions > 1.70 g/ml pooled for subsequent metagenomic sequencing. Each point represents an average of four biological replicates.

Pooled volumetric fractions from heavy DNA (Figure 1) from density-gradient centrifugation were used to generate an Illumina sequence library with an average insert size of 400 bp that was sequenced on an Illumina NextSeq 500 with paired-end 150 bp reads at the DNASU Core Facility at Arizona State University. The metagenomic sequencing produced an average of 49.4M reads for biochar-amended samples and 40.9M reads for control samples. Estimates of average coverage and sequence diversity for each metagenomic data set were carried out with Nonpareil 3 using default settings (Rodriguez-R and Konstantinidis, 2014; Rodriguez-R et al., 2018). The raw sequencing reads were quality filtered and trimmed to remove Illumina adaptors using Trimmomatic version 0.36 (Bolger et al., 2014), paired-end reads were interleaved using the interleave-reads.py script from khmer version 2.1.1 (Crusoe et al., 2015) before assembly. The assembly of metagenomes was carried out using SPAdes version 3.11.1 (Bankevich et al., 2012) with default parameters and the kmer list: 27, 37, 47, 57, 67, 77, 87. The assembled contigs were quality checked by mapping the raw reads to contigs using Bowtie2 version 2.2.5 (Langmead et al., 2009; Langmead and Salzberg, 2012). SAMtools version 1.8 (Li et al., 2009) was used to sort and index the mapping files and extract contig coverage information. Coverage of the assembled contigs was calculated using BEDTools2 version 2.24.0 (Quinlan and Hall, 2010), assembled contigs quality filtered to remove contigs with <90% coverage. The quality of the filtered assemblies was assessed with QUAST version 3.0 (Gurevich et al., 2013).

The mapping data and coverage information were used to bin contigs into population genome bins separately for each ^13^C-metagenome with MetaBat version 2.12.1 (Kang et al., 2015) using a minimum contig length of 2000 bp. CheckM version 1.0.11 (Parks et al., 2015) was used to evaluate the level of bin completeness and contamination based on domain-level single-copy genes. Genome bins (i.e., MAGs) with over 50% completion according to CheckM were imported in to Anvio version 6.1 (Eren et al., 2015) to be manually curated, which typically improved bin quality by reduction of contamination level. The quality of refined MAGs was assessed by running CheckM and MAGs with >50% completeness and <10% contamination were used for downstream analysis.

### Metagenomic Annotation and 16S rRNA Gene Analysis

Taxonomic classification for each MAG was carried out using GTDB-Tk (Chaumeil et al., 2019) against the Genome Taxonomy Database (GTDB) (Parks et al., 2018, 2019). MAG abundance was calculated using the “bin_coverage_individualassembly.pl” script^2^. For the assessment of taxonomic composition of each metagenome, 16S rRNA gene fragments were first recovered from metagenomes using Barrnap version 0.9^3^. Average coverage of 16S rRNA gene fragments were determined by mapping metagenomic libraries using Bowtie2 version 2.2.5 (Langmead et al., 2009; Langmead and Salzberg, 2012) and mean depth calculated using the jgi_summarize_bam_contig_depths script in MetaBat version 2.12.1 (Kang et al., 2015). To assess community structure, Barrnap output sequences were parsed to remove 23S and 5S gene fragments then input in the RDP classifier (Wang et al., 2007) with confidence cutoff of 80%. The resulting output was used to generate a Bray–Curtis distance matrix in Rstudio v. 3.3.2 using the phyloseq package (McMurdie and Holmes, 2013). Protein-coding genes within the MAGs were identified using Prodigal version 2.6.3 (Hyatt et al., 2010) and functional annotation was carried out using GHOSTKOALA (Kanehisa et al., 2016). We specifically focused on the effect of each treatment on the presence or absence of genes for the catabolic processes of various C-complexes with different decomposability, ranging from the highly recalcitrant aromatic compounds to the more labile monosaccharides, sugar acids and sugar alcohols, more attention was also given to the genes for N metabolism. Statistics were performed using Rstudio v. 3.3.2 with general dependency on the following packages: ggplot2 (Wickham, 2009), dplyr (Wickham et al., 2019), cowplot (Wilke, 2017) and reshape2 (Wickham, 2007). The vegan R-package (Oksanen et al., 2018) provided tools to calculate non-metric multi-dimensional scaling on Bray-Curtis distance matrix (vegdist) and significant differences in the functional and taxonomic communities between treatments were tested with permutational analysis of variance (PERMANOVA) (Anderson and Walsh, 2013) and analysis of similarity (ANOSIM) (Clarke et al., 2008). To identify genes that were differentially present between active community of control and biochar-amended samples, DESeq2 package was employed (Anders and Huber, 2010). A count table of functional annotations was generated using the Kyoto Encyclopedia of Genes and Genomes (KEGG) orthology (KO) terms. Each column represented a metagenome and each element was the count of reads from the metagenome assigned to the KO term. DESeq2 was used with default settings to estimate the effective size library and variance to normalize the counts prior to the detection of difference between biochar-amended and control metagenomes for each KO term.

## Results

### Enrichment of ^13^C-DNA and Statistics of Metagenomes

Our study focused on soils collected 2 years after the initial addition of biochar to an Oxisol soil under napiergrass cultivation (Yu et al., 2018). Eight samples from the biochar-amended and control plots, collected prior to harvest, were used to determine soil chemical characteristics. Between plots, few exhibited significant differences in elemental concentration and nutrient status (Supplementary Table S1). Mean C concentration (C%) of biochar-amended soils was previously shown to be significantly higher compared to soils from control plots (Yu et al., 2019). Similar to our previous findings, no statistical differences between soil plots were observed in soil base cations (calcium [Ca^2+^], sodium [Na^–^], magnesium [Mg^2+^], and potassium [K^+^]), pH or total nitrogen concentration. Comparison of the active community between biochar-amended and control soil microcosms was based on the respiration, or cumulative gas production. The rate of CO_2_ production was not significantly different between biochar-amended and control soil microcosms (Supplementary Figure S1). After 14 days of incubation, CO_2_ concentration comprised approximately 15% of the headspace. In our previous comparative metagenomic study, we found significantly higher copies of genes involved in denitrification (Yu et al., 2019), therefore in the current experiment we measured N_2_O gas to further explore this. However, no significant difference was found between N_2_O production rates in the biochar-amended and control microcosms (Supplementary Figure S1).

Total DNA concentrations were measured in 23 density gradient fractions to detect buoyant density shifts after the mineralization of ^12^C- or ^13^C-labeled perennial ryegrass after 14 days. The heavy density fractions had a buoyant density between 1.70 and 1.717g ml^–1^. In the control soil microcosms the heavy density fractions contained between 58-times to 2.9-times more DNA than the fractions from the soil microcosms that contained the ^12^C- labeled perennial ryegrass. Biochar-amended soil microcosms heavy density fractions contained between 166.8-times to 16.4-times more DNA than the DNA fractions from the soil microcosms that contained the ^12^C- labeled perennial ryegrass (Figure 1). To further confirm the enrichment of ^13^C-labeled DNA, the 16S rRNA gene was quantified in density fractions between 1.683 and 1.718 g ml^–1^. The heavy density-gradient factions with buoyant density ranging from 1.701 to 1.711g ml^–1^ contained over 100-times more 16S rRNA gene copies in ^13^C-incubated samples than in the ^12^C-controls for both control and biochar-amended microcosms (Figures 1A,B). Heavy gradient fractions from biochar-amended and control microcosms containing at least 100-times higher levels of ^13^C incorporation were thereafter pooled for each microcosm for subsequent shotgun metagenomic sequencing.

^13^C-labeled DNA was sequenced from four replicate samples representing the biochar-amended soils (Plots 1, 3, 4, 8) and four samples representing the control soils (Plots 2, 5, 6, 7), yielding approximately 24–53 megabytes of short paired-end sequence data per sample. The Nonpareil algorithm that was used to estimated coverage based on the read redundancy value revealed an average coverage of approximately 0.65 and 0.54 for metagenomes obtained from biochar-amended and control Oxisol samples, respectively (Supplementary Figure S2). On average, the coverage of the DNA-SIP metagenomes (0.60, this study) was much higher compared to our previous study using whole community metagenomes (0.32, Yu et al., 2019) of the same soils. The sequence diversity values, a measure of alpha-diversity derived from Nonpareil curves, exhibited no differences between the biochar-amended (average, 21.23) and control soils (average, 21.04). Metagenomes from ^13^C-labeled biochar-amended and control soil were assembled and quality checked to produce contigs for binning. The quality assembled contigs in biochar-amended and control metagenomes amounted to 361,137 and 258,035 bp within contigs longer than 1.5 kb, respectively. The N_50_ values averaged 580 and 592 bp from biochar-amended and control metagenomes. Sequence statistics for each treatment plot were summarized in Table 1.

**TABLE 1:** Metagenomic sequence and assembly summary.

				Nonpareil		SPADES Assembly		
Samples	Treatment	No. Reads	Trimmed Reads	Coverage (%)	Diversity	No. Contigs	N50	Longest Contig
Plot 1	Biochar-amended	48,939,158	43,886,293	62.02	21.59	1,685,259	731	214,999
Plot 3	Biochar-amended	53,550,803	49,275,476	70.64	20.78	2,573,173	604	116,946
Plot 4	Biochar-amended	45,680,993	41,882,029	63.85	21.15	2,601,521	505	127,320
Plot 8	Biochar-amended	49,575,137	45,710,094	64.48	21.40	2,841,923	480	290,581
Plot 2	Control	49,463,602	45,320,132	70.67	20.88	2,302,470	503	651,253
Plot 5	Control	48,647,720	44,555,996	69.83	20.64	2,381,807	491	137,757
Plot 6	Control	24,655,227	22,260,113	9.22	21.20	1,528,627	453	179,238
Plot 7	Control	41,014,445	37,866,468	65.91	21.44	1,002,610	922	102,465

### Active Community Taxonomic Composition and Functional Diversity

**FIGURE 2:**
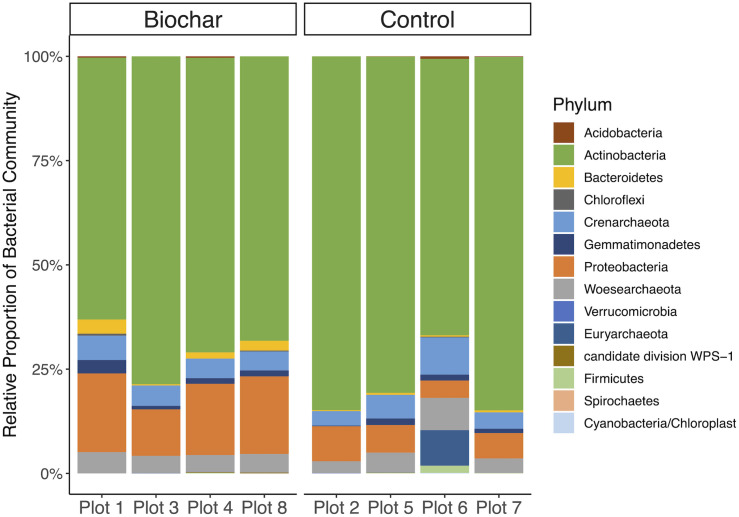
Taxonomic affiliation of recovered 16S rRNA gene fragments. Mean relative abundance of bacterial phyla for each plot for biochar-amended and control treatments. Underlying data is based on average coverage depth of 16S rRNA gene-encoding fragments recovered from metagenomic datasets.

The taxonomic affiliations of recovered 16S rRNA gene fragments (metagenome-derived) showed little to no differences between the biochar-amended and control metagenomes (Figure 2). The active bacterial community in biochar-amended and control metagenomes were primarily represented by the phyla *Actinobacteria* at 71.5 and 80.0%, followed by *Proteobacteria* at 15.7 and 6.5%, respectively (Figure 2). The next most abundant bacterial phyla in the biochar-amended metagenomes were the *Bacteroidetes* and *Gemmatimonadetes* which represented 1.6 and 1.5% of the community, respectively. In the control metagenomes, *Gemmatimonadetes* composed 1.0% of the community and *Bacteroidetes* represented 0.4%. The remaining bacterial phyla represented less than 1% of the community. The active archaeal phyla in the biochar-amended and control metagenomes were represented by *Crenarchaeota* at 4.9 and 5.3%, followed by *Woesearchaeota* at 4.4 and 4.5%, of the community respectively. In the control metagenomes, *Euryarchaeota* represented 1.7% of the community, but were <1% in the biochar-amended metagenomes.

**FIGURE 3:**
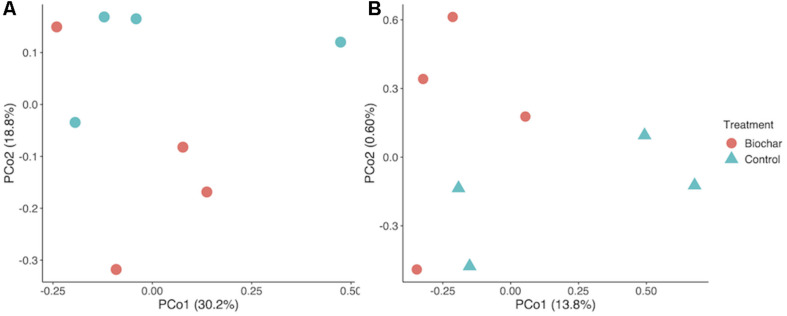
Taxonomic and functional shifts as an effect of biochar amendment. **(A)** PCoA plot of taxonomic community composition. **(B)** Principle coordinate analysis (PCoA) Plot of KO term annotations. Underlying data are based on Bray–Curtis distance matrix derived from a KO term count matrix. Underlying data are a Bray–Curtis distance matrix of 16S rRNA gene-encoding fragments recovered with Barrnap and processed in the RDP classifier.

Biochar had no significant effect the relative abundance of most bacterial phyla, though *Proteobacteria* and *Bacteroidetes* were significantly enriched in the biochar-amended metagenomes (two-tailed *t*-test, *P* < 0.05). Biochar also did not cause a significant shift in taxonomic β-diversity, based on Bray–Curtis distances of phylum-level active community composition (ANOSIM, *P* = 0.66) (Figure 3A). In addition, biochar amendment generally did not significantly shift the functional gene content of the active community, summarized as KO terms (ANOSIM, *P* = 0.16) (Figure 3B). However, of the 6097 KO terms with abundances adequate for *P*-value assignment in DESeq2, three KO terms differed significantly (adjusted *P* < 0.05) and another eight differed nearly significantly (adjusted *P* < 0.1) between control and biochar-amended metagenomes. DESeq2 analysis revealed a statistically significant decrease in nitrate reductase abundance (KO00370 *narG*; *narZ*; *nxrA*) in biochar-amended metagenomes and a significant enrichment of genes involved in bacterial motility-pilus systems and type VI secretion systems (Supplementary Table S2).

### Recovery of MAGs and Diversity of MAGs Involved in C and N Cycling

To precisely identify and quantify individual populations in the active community, we performed genome binning analysis of the individually assembled metagenomic data sets. Between 20 and 48 bins were recovered through binning for each individual metagenome. Due to low completeness of some MAGs, a threshold of 50% completeness and less than 10% contamination, based on the presence of 71 single-copy bacterial genes, was established for further analysis. These medium- to high-quality MAGs collectively recruited about 4.11 and 4.81% of the short reads, on average, for the both^13^C-biochar-amended and ^13^C-control, respectively. After refining genomic bins, 84 population MAGs (49 and 35 MAGs from ^13^C-biochar-amended and ^13^C-control) remained, these represented ∼25% of the total MAGs obtained. Assigned taxonomies at the family-level and genomic characteristics of genome bins used in this study are summarized in Table 2 (Supplementary Table S3). Genome size ranged from 1.84 to 11.9 Mbp, and G+C% content varied from 58.3% to 73.1% (Supplementary Table S3). Inferred taxonomy revealed that MAGs recovered from the active community represented members of *Acidobacteria*, *Actinobacteria*, *Gemmatimonadetes*, and *Proteobacteria (Alphaproteobacteria, Betaproteobacteria*, and *Gammaproteobacteria)* in both soil metagenomes, whereas *Myxococcota* (*Deltaproteobacteria*) were characteristic of biochar-amended soils (Supplementary Table S3).

**TABLE 2:** Taxonomic classification and characteristics of genome bins that were at least 80% completeness calculated from CheckM.

Genome Bin I.D.	Average Bin^*a*^ Coverage	Taxonomy^*b*^ (Family-level)	Completeness (%)	Contamination (%)	GC (%)	Size (Mbp)	Coding Density
**Biochar-amended soil**							
Bin.1_22	16.18	Dermatophilaceae	89.8	3.88	71.8	3.68	91.72
Bin.1_3	8.40	20CM-4-69-9	84.2	3.74	70.1	3.79	93.92
Bin.1_36	26.46	Gemmatimonadaceae	90.7	2.75	69.9	3.77	92.86
Bin.3_15	8.74	Burkholderiaceae	80.0	2.52	68.0	4.66	88.90
Bin.3_19	29.67	Streptomycetaceae	88.7	6.45	71.0	10.9	89.54
Bin.3_21	9.38	Gemmatimonadaceae	83.2	3.85	70.2	3.68	91.43
Bin.3_29	18.84	Micromonosporaceae	80.0	4.30	70.1	7.30	91.71
Bin.3_38	23.85	Gemmatimonadaceae	89.8	2.75	69.9	3.73	92.75
Bin.3_9	14.00	Rhizobiaceae	91.1	3.42	63.1	5.06	88.75
Bin.4_17_1	41.92	Micromonosporaceae	82.5	6.25	70.2	6.73	92.29
Bin.4_3	12.46	Gemmatimonadaceae	85.7	4.4	69.6	4.27	91.77
Bin.4_31	11.82	Gemmatimonadaceae	88.1	7.74	70.3	3.54	91.90
Bin.4_30_1_1	12.04	Rhodanobacteraceae	92.1	0.94	69.2	3.14	90.43
Bin.8_14	20.20	Haliangiaceae	90.5	3.39	68.3	9.81	93.72
Bin.8_16	17.27	Polyangiaceae	94.9	5.18	66.2	11.9	91.83
Bin.8_36	34.44	Micromonosporaceae	86.2	3.28	70.3	7.13	92.28
Bin.8_40	15.16	Dermatophilaceae	87.1	3.80	71.7	3.54	91.67
Bin.8_42	11.74	Gemmatimonadaceae	89.9	2.75	70.2	4.10	91.37
Bin.8_6	31.06	Gemmatimonadaceae	90.5	2.2	69.9	3.75	92.85
**Control soil**							
Bin.2_24	26.22	Micromonosporaceae	96.5	1.93	69.1	7.45	91.71
Bin.2_3	11.48	QHCE01	95.3	1.26	58.5	3.05	90.84
Bin.2_7	13.36	Sphingomonadaceae	89.0	8.83	64.4	2.29	93.71
Bin.5_1	12.81	Nocardioidaceae	90.8	5.04	72.5	4.71	92.4
Bin.5_19	15.38	Gemmatimonadaceae	92.0	2.75	70.2	4.04	91.54
Bin.5_27	12.34	Micromonosporaceae	83.7	6.06	71.3	4.26	91.08
Bin.5_5	28.65	Gemmatimonadaceae	90.7	2.75	69.9	3.77	91.91
Bin.6_1_1	21.92	Streptomycetaceae	85.8	9.65	70.0	11.0	87.19
Bin.6_18	7.72	Sphingomonadaceae	90.5	4.27	64.9	2.34	93.07
Bin.6_3	8.27	Acidobacteriaceae	90.3	1.94	58.3	5.17	89.72
Bin.6_6	12.51	Gemmatimonadaceae	85.9	4.72	69.6	3.81	92.05
Bin.6_9	75.61	Catenulisporaceae	88.5	3.86	70.9	9.53	90.18
Bin.7_12	30.29	Streptosporangiaceae	81.5	7.75	71.3	9.15	92.13
Bin.7_13	29.74	Gemmatimonadaceae	88.7	2.20	69.9	3.71	92.85
Bin.7_20	23.97	Dermatophilaceae	89.8	5.89	71.7	3.72	91.77
Bin.7_21	12.05	Burkholderiaceae	87.5	0.31	68.0	4.83	88.69

*^a^Bin coverage is calculated using perl script. Bin coverage is weighted by the length. ^b^Taxonomy determined using GTDB-Tk at the family level.*

**FIGURE 4:**
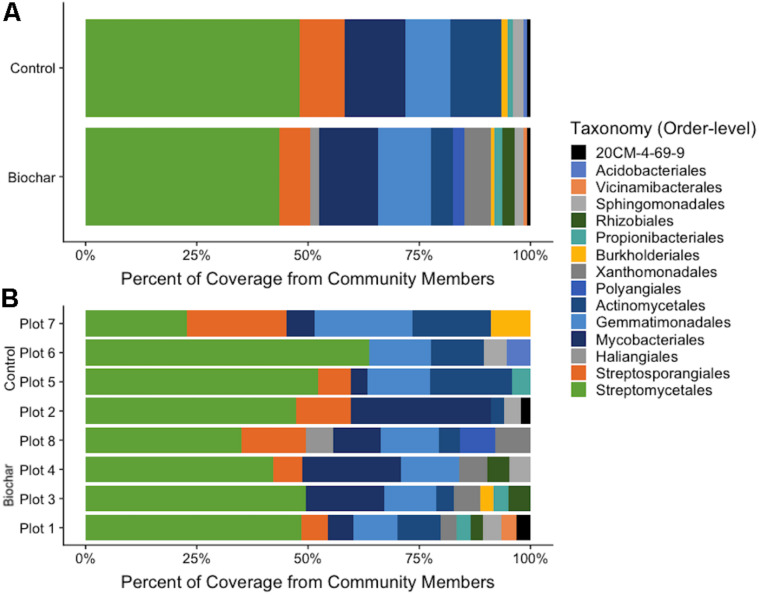
Proportion of abundance of recovered populations from metagenomes. **(A)** Proportion of MAG abundance from biochar-amended and control metagenomes. **(B)** Proportion of MAG coverage from each plot. Abundance was calculated as bin coverage normalized by contig lengths. Taxonomic classification is based on GTDB-Tk database.

Estimates of MAG abundance within a metagenome was calculated by normalizing average bin coverage by the contig lengths. The majority of recovered MAGs in both the ^13^C-biochar-amended and ^13^C-control metagenomes belonged to phylum *Actinobacteria* (Figure 4), represented by *Actinomycetales* (avg. coverage of metagenome: 5% biochar-amended, 11.6% control), *Mycobacteriales* (avg. coverage of metagenome: 13.3% biochar-amended, 13.7% control), *Propionibacteriales* (avg. coverage of metagenome: 1.7% biochar-amended, 1.4% control), *Streptosporangiales* (avg. coverage of metagenome: 7% biochar-amended, 10.1% control), 20CM-4-69-9 (avg. coverage of metagenome: 0.8% biochar-amended, 0.8% control), and *Streptomycetales* (avg. coverage of metagenome: 43.6% biochar-amended, 48.1% control) (Figure 4). Approximately 20% (10/49) of MAGs recovered from ^13^C-biochar-amended and ^13^C-control soils were taxonomically assigned at the order-level to *Streptomycetales*. The next most abundant MAGs recovered were assigned to phylum *Gemmatimonadetes* which represented an average coverage of 11.9% (7 MAGs) and 10% (5 MAGs) of ^13^C-biochar-amended and ^13^C-control metagenomes, respectively (Figure 4A). MAGs assigned to phylum *Proteobacteria*, represented by order *Burkholderiales* (avg. coverage of metagenome: 0.8% biochar-amended, 1.3% control) and *Sphingomonadales* (avg. coverage of metagenome: 1.8% biochar-amended, 2.2% control), were recovered from both ^13^C-biochar-amended and ^13^C-control metagenomes. Proteobacterial orders *Rhizobiales* and *Xanthomonadales* MAGs were only recovered from the biochar-amended metagenomes and comprised on average 2.9 and 0.8% coverage of the ^13^C-biochar-amended metagenomes, respectively. Additionally, the *Myxococcota* were only recovered from one ^13^C-biochar-amended metagenomes, further classified as *Haliangiales* and *Polyangiales* with 1.9% and 2.4% of coverage of the Plot 8 metagenome, respectively (Figure 4B). *Acidobacteria* MAGs recovered from ^13^C-biochar-amended and ^13^C-control metagenomes were assigned to order Vicinamibacterales and *Acidobacteriales*, respectively, and represented 0.9% of the coverage their respective metagenome (Figure 4).

**FIGURE 5:**
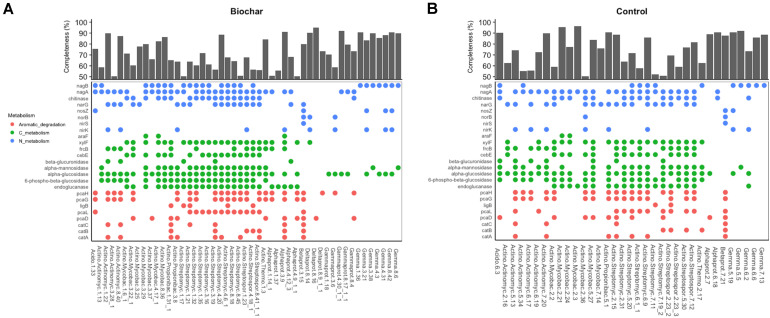
Metabolic features of medium- and high-quality MAGs recovered from biochar-amended and control metagenomes. **(A)** Presence/absence of gene in MAGs recovered from biochar-amended metagenomes and completeness of biochar MAGs and taxonomic classification at phylum-level. **(B)** Presence/absence of gene in MAGs recovered from control metagenomes and completeness of control MAGs and taxonomic classification at phylum-level.

The examination of agriculturally relevant N-cycling genes showed that 46 MAGs, representing different bacterial phyla, possessed at least one gene involved in denitrification. Genes involved in nitrification and nitrogen fixation were not observed in the recovered MAGs. Among MAGs that possessed denitrification genes, 25 and 21 MAGs were recovered from ^13^C-biochar-amended and ^13^C-control metagenomes, respectively. MAGs obtained from both soils contained a gene involved in single steps of the denitrification pathway. Those that contained genes involved in denitrification generally belonged to the phyla *Actinobacteria* and *Proteobacteria*. Of the 34 *Actinobacteria* MAGs that possessed denitrification genes, four MAGs recovered from biochar-amended soils and six MAGs recovered from control soils contained at least one copy of *nirK* and *narG* genes (Figures 5A,B). Nearly all MAGs assigned to *Streptomycetales* and *Streptosporangiales* had at least one copy of *narG*. Two *Alphaproteobacteria* MAGs recovered biochar-amended soil contained one copy of either *nirK* or *narG* genes, however, *Alphaproteobacteria* MAGs recovered from the control soil did not possess denitrification genes. Both *Burkholderiales* MAGs possessed all necessary genes to perform complete denitrification (i.e., reduction of NO_3_^–^ or NO_2_^–^ to N_2_) (Figure 5). At least one copy of *nirK* and *norB* genes were found in a *Gammaproteobacteria* (Bin3.6) and *Deltaproteobacteria* (Bin8.14) MAG recovered from biochar-amended soil. Nearly half of *Gemmatimonadetes* MAGs contained a copy of *nirK*, of these four MAGs, all recovered from biochar-amended metagenomes, also contained a copy of *nosZ*. In addition, the *Acidobacteria* MAG recovered from the biochar-amended soil (Bin 1.33) possessed the *nosZ* gene, while nosZ was not observed in the Acidobacteria MAG recovered from the control metagenomes. Other N-cycling genes observed in recovered MAGs included a gene for a cytochrome c nitrite reductase (*nrfA*) associated with dissimilatory nitrate reduction to ammonium (DNRA), which was found in two MAGs recovered from biochar-amended metagenomes (Bin1.21 and Bin8.9_1_1). All recovered *Gemmatimonadetes* MAGs also possessed *nagB* (ammonia from amino sugars) (Figures 5A,B).

In addition to N-cycling, we sought to identify genes in the recovered MAGs that encode for enzymes directly involved in the decomposition of plant organic carbon via hydrolysis of glycosidic bonds that target cellulose (e.g., endoglucanases), hemi-cellulose (e.g., xylanases), cellobiose (e.g., beta-glucosidase), and ring-opening enzymes involved in degradation of aromatic compounds prevalent in soils. Most *Gemmatimonadetes* MAGs contained genes involved in degradation of labile carbohydrates such as endoglucanase, alpha-glucosidase and alpha-mannosidase but did not have genes for aromatic degradation (Figures 5A,B). On the other hand, both Betaproteobacteria (i.e., *Burkholderiales*) MAGs possessed key genes in the beta-ketoadipate pathway including genes for catechol ortho-cleavage to 3-oxoadipate (i.e., *catABC* and *pcaDL*) and the ring-opening step of protocatechuate degradation (i.e., *pcaGH*). However, genes for the degradation of more labile compounds were not observed in these MAGs. *Alphaproteobacteria* and *Gammaproteobacteria* MAGs possessed genes involved in a single step of the beta-ketoadipate pathway and genes associated with degradation of labile carbohydrates and sugar transport systems. Overall, Actinobacteria MAGs possessed multiple copies of gene associated with the degradation of plant biomass C, such as cellulose, hemi-cellulose and cellobiose (i.e., endoglucanases, beta-glucosidases, alpha-glucosidases, alpha-mannosidase), binding proteins involved in sugar transport, ring-opening enzymes or a partial beta-ketoadipate pathway (Figures 5A,B).

## Discussion

### Biochar Had Negligible Effects on the Active Soil Community

In this study, we examined the active community of a tropical Oxisol 2 years after the initial addition of biochar under napiergrass cultivation. We investigated the impact of biochar amendment on the genomic diversity and functional potential of active soil bacterial community using DNA-SIP shotgun metagenomics and MAGs (see below). Here, we did not observe a significant shift in the composition of the active community in response to biochar. This finding contrasts with our previous study, based on 16S rRNA amplicon analysis, which found significant changes in the community composition and alpha diversity in response to a month and a year after biochar addition (Yu et al., 2018). However, this is consistent with our previous comparative metagenomic study on the total community, which showed that biochar did not have a significant effect on the community composition 2 years after biochar addition (Yu et al., 2019). Altogether, this may indicate that the soil microbial community is resilient to a single biochar addition as we were unable to detect compositional changes in the whole and active community as time from the initial disturbance increased.

The strength of environmental disturbances and the frequency it is applied can have an effect on the resilience of the microbial composition (Allison and Martiny, 2009). Here and in our previous studies, a low-volatile matter biochar was only added at the initiation of the experiment. In addition, the microbial community was initially sensitive to perturbations related to the addition of biochar but the strongest determinant of the community composition was soil type, as well as the degree of biochar-related changes in composition determined by soil type (Yu et al., 2018). Although examination of 16S rRNA gene fragments extracted from each individual metagenome did show a significant increase in *Proteobacteria* and *Bacteroidetes* abundance in the biochar metagenomes, it is important to note that the number of recovered 16S rRNA genes per metagenome was extremely low compared to the number of gene-encoding sequences. This may reflect difficulties presented by the massive data volume of metagenomes, high sequence similarity of 16S rRNA genes and skewed species abundance which make rRNA recovery from metagenomic datasets difficult (Yuan et al., 2015).

In addition, the similarity of the active communities between biochar-amended and control soils may reflect the conditions of the experimental set up. For instance, sieved soils resulting in different size fractions have been shown to support distinct microbial communities (Bach et al., 2018; Fox et al., 2018) and the input of fresh organic matter has been shown to stimulate a select group of bacteria (Pascault et al., 2013). Previous studies using ^13^C-DNA-SIP showed that the C assimilating bacterial phyla found in heavy-fraction soil DNA enriched with maize and wheat residue were primarily distributed among phyla *Actinobacteria, Proteobacteria*, and *Firmicutes*, and the quality of plant material has a strong influence on the composition of the degrading communities (Bernard et al., 2007; Pascault et al., 2013; Fan et al., 2014; Su et al., 2017). Here, the major bacterial phyla recovered from the active community were primarily distributed among known plant biomass degrading *Actinobacteria* suborders, such as *Actinomycetales, Streptomycetales, Propionibacteriales, Mycobacteriales*, with genomes known to be particularly enriched in carbohydrate-active enzyme genes (Lewin et al., 2016).

Previous studies have examined how short- and longer-term biochar application affects soil communities (Anders et al., 2013; Noyce et al., 2015; Jenkins et al., 2017; Zhang et al., 2019). They principally revealed that biochar amendment is accompanied by significant shifts in soil chemistry and the soil microbial community. Other studies have observed negligible biochar effects on soil community structure, GHG production, and plant productivity or that biochar effects were transient and showed no long-term effects (1–3 years) on microbial growth rates in agricultural soils (Rousk et al., 2013; Meschewski et al., 2019; Azeem et al., 2020). Our results concur with the latter in that biochar amendment did not significantly shift the active taxonomic or functional communities, at least over a period of 2 years.

These findings contradict the results of our previous study on the same soils (Yu et al., 2019), which observed significantly higher relative abundances of *Proteobacteria* and *Bacteroidetes* and an enrichment of genes involved in pathways, such as denitrification, respiration and metabolism of aromatic compounds with biochar amendment. We also failed to find support of our initial hypothesis, based on our earlier findings, that biochar would have a significant positive effect on genes involved in denitrification in the active microbial community. The results of our current study showed that the *narG* gene, encoding nitrate reductase, was significantly higher in the control soil metagenomes, while no significant differences were observed for other denitrification genes, such as *nirK*/*nirS*, *norB*, and *nosZ*. This was not expected since previous studies focused on biochar effects on denitrification found that biochar increased the abundance of nitrite reductase genes (*nirK*/*nirS*) (Ducey et al., 2013; Liu et al., 2018), and nitrous oxide reductase genes (*nosZ*) (Xu et al., 2014; Harter et al., 2016) in soil. Overall, our results showed that biochar addition did not affect the active denitrifying community, which may suggest that long-term effects of biochar application do not alter the potential for microbially-mediated N loss from these agricultural soils.

We also observed a significant increase in genes for an outer membrane usher protein (*fimD*) and type VI secretion system protein (*impL*) associated with biochar, which may indicate bacterial movement and communication (Gallique et al., 2017; Yang and Dirk van Elsas, 2018). However, whether this is impacted by biochar or reflect indirect effects of the microcosm experiment remain unresolved and outside the scope of this study. Based on these results we conclude that even if the agricultural application of biochar impacts the soil microbial community in the short-term the effects are not lasting in the active community. These findings may suggest that the active soil microbial community is functionally resilient to a single biochar application, and biochar effects may be overwritten by the other factors, such as land management or cropping system (Hardy et al., 2019; Azeem et al., 2020).

### Recovery of Populations of the Active Community

By coupling SIP with shotgun metagenomics we selected for the active community and improved resolution within the high diversity environment of the soil and demonstrated the ability to assemble several high quality MAGs. By assembling MAGs of active populations, we have gained a new depth of insight into the putative nutrient cycling and life strategies of some Oxisol agricultural soil microorganisms that was not possible with only metagenomics. We recovered 12 *Gemmatimonadetes* MAGs from ^13^C-DNA with an average coverage of about 11%, which composed a higher proportion of the active community than expected. *Gemmatimonadetes* were better represented in the recovered MAGs than compared to our previous studies based on 16S rRNA amplicon and rRNA gene fragments (metagenome-derived), which composed approximately 1–1.5% of the Oxisol soil communities (Yu et al., 2018, 2019). This contrast with other SIP studies, which have generally recovered *Gemmatimonadetes* sequences from the unlabeled light fraction, suggesting this group may be oligotrophic and likely correspond to K-strategies (Bernard et al., 2007; Pascault et al., 2013). Our finding highlights that low abundant soil bacteria can be fast-growing (i.e., sufficient growth within 14 days) and metabolically versatile. For instance, *Gemmatimonadetes* MAGs encoded the genes involved in labile C (e.g., starch) metabolism and organic N cycling (e.g., *N*-acetyl glucosaminidase). In addition, four of the *Gemmatimonadetes* MAGs encoded enzymes necessary for the reduction of NO_2_ and N_2_O (i.e., *nirK* and *nosZ*), which is consistent with studies that have reported *Gemmatimonadetes* as *nirK* denitrifiers and have N_2_O reduction ability (Helen et al., 2016; Park et al., 2017). Interestingly, this finding contrasts somewhat with earlier studies that have previously shown that the genetic linkage of *nirS* and *nosZ* is the predominant pattern of denitrification genes (Graf et al., 2014). This may suggest that less abundant microorganisms may play an important role in increasing functional redundancy, which can enhance the ability of soil communities to counteract environmental disturbances. The functional importance of low-abundant microbes may be due to effects that are disproportionately large given their abundance (i.e., keystone species) or as a provision of the insurance effect, that rare or low abundance species may offer a pool of genetic resources that can be activated when the appropriate conditions are met (Jousset et al., 2017). Shade et al. (2014) estimated that conditionally rare taxa, those which are rare in most conditions but become dominant occasionally, comprised 1.5–28% of all microbes.

In both the biochar-amended and control metagenomes, MAGs that contain a nitrite reductase gene predominantly harbored *nirK*, which encodes the copper-containing nitrite reductase. The cytochrome cd_1_ nitrite reductase encoded by the *nirS* gene was only found in the *Burkholderiales* MAGs, which also were the only MAGs that encoded all enzymes required to perform complete denitrification. Variable abundance ratios of *nirK* and *nirS* genes have previously been reported, with a trend of *nirK* abundances to be more sensitive to nutrient changes and higher in bulk soil, and *nirS* abundance to be higher in the rhizosphere (Henry et al., 2004; Kandeler et al., 2006; Bárta et al., 2010). This would be consistent with our soil collection (i.e., bulk soil) although we did not observe significant differences in soil chemical data. These findings are consistent with previous studies that have proposed a modular assembly for denitrification pathways in soils and suggested shared regulatory mechanisms that may constrain the loss of *nor* and *nos* in *nirS*-type denitrifiers (Graf et al., 2014; Orellana et al., 2014). In addition, nine MAGs related to several actinobacterial suborders had *nirK* and *narG* genes, though whether there are co-occurrence patterns between *nirK* and *narG* remain unclear. In fact, majority of *Actinobacteria* MAGs from both metagenomic datasets contained at least one copy of *narG* gene. Earlier studies of *narG* diversity in soil environments have identified sequences related to those from *Actinobacteria* (Philippot et al., 2002; Palmer and Horn, 2012), which may highlight the importance of *Actinobacteria* in the nitrate reducing community of Oxisol soils. Altogether, these findings highlight the importance of accounting for the different organisms and their interactions to better understand denitrification processes in soils, as the reduction of nitrate or nitrite to N_2_ would require the combined participation of different N-reducing bacteria.

We explored the impact of active microbial populations in the potential breakdown and recycling of plant biomass in soils, by surveying genes associated with biomass and aromatic degradation in the recovered MAGs. Overall, the *Actinobacteria* MAGs encoded the greatest number and variety of enzymes involved in the degradation of plant biomass, which was expected since this taxonomic group has many representatives that have been characterized for their ability to degrade a variety of labile and recalcitrant organic compounds. For example, *Actinomycetes* can compete with fungi for lignin degradation (De Boer et al., 2005), Mycobacteria can degrade polycyclic aromatic hydrocarbons under oligotrophic conditions (Uyttebroek et al., 2006), and aerobic cellulose degradation has been demonstrated by a number of *Actinobacteria* species (Anderson et al., 2012). In the biochar-amended soil metagenomes we recovered more diverse *Proteobacteria* MAGs, which was not surprising due to the higher amount of total C% in the biochar-amended soils. The *Rhizobiales* MAGs contained the genetic potential to degrade a variety plant organic C and complete or partial β-ketoadipate pathway, similar to many related *Rhizobiales* (MacLean et al., 2006). The β-ketoadipate pathway is present in many members of the *Rhizobiaceae* family, emphasizing the importance of aromatic acid catabolism in this family (Parke and Ornston, 1986). In addition to the denitrification pathway, *Burkholderiales* MAGs also had the complete set of genes in the β-ketoadipate pathway but did not have gene for degradation or transport of labile plant C compounds. This finding was not surprising as the representative of the *Burkholderiales* order (genus *Cupriavidus*) have been reported to degrade recalcitrant C and aromatic compounds including lignin (Shi et al., 2013) and phenoxy herbicides (Cuadrado et al., 2010). From a functional point of view, the active population was composed of a small diversity of species that harbored different degradation capabilities, which may suggest the different trophic behaviors with copiotrophic *Actinobacteria* (Ho et al., 2017) and *Rhizobiales* (Bastida et al., 2015) and oligotrophic *Acidobacteria* (Fierer et al., 2007; Ho et al., 2017) and *Burkholderiales* (Nicolitch et al., 2019) with the genetic potential to degrade labile (e.g., cellulose and hemicellulose) or more refractory compounds like aromatics, respectively.

## Conclusion

The combination of metagenome and MAG analysis allowed for an increased understanding of the potential biological functions of the active soil microbial community as altered by biochar addition. Potential carbon cycling pathways in both datasets appeared to not be significantly altered by biochar, especially related to complex carbon sources. In addition, we observed no difference in genes involved in the denitrification pathway. However *narG* was significantly higher in the control metagenomes of the active communities. In summary, this study demonstrated the application of DNA-SIP combined with shotgun metagenomics and genomic binning to identify active populations in a tropical Oxisol soil under biochar amendment. The results indicated that the taxonomic and functional composition of the active community was not significantly affected by biochar amendment. Finally, we were able to recover high quality MAGs of low-abundance populations using DNA-SIP to target the active community. These results may suggest that application of biochar may influence the microbial communities and their function soon after application, however, the effects on the microbial community are not lasting. Although biochar did not have lasting effects on the active soil community, it may still be a promising strategy for the intended purpose of biochar in agricultural soil. For example, biochar addition can result in long-term sequestration of C without a significant long-term influence on the soil microbial community which may lead to unexpected nutrient losses from the soil through biotic processes, such as denitrification.

## Data Availability Statement

The datasets for the biochar-amended and control metagenomes raw sequences and assembled MAGs can be found in GenBank deposited under PRJNA622594. This data can be found here: (https://www.ncbi.nlm.nih.gov/bioproject/PRJNA622594).

## Author Contributions

JY conducted the soil incubations and library preparation and wrote up the manuscript. JY, MP, and CP analyzed the data and discussed the results. LD, SC, and JD designed the field experiment, collected the soil samples, and carried out the chemical characterization of soils. All the authors contributed to the article and approved the submitted version.

## Conflict of Interest

The authors declare that the research was conducted in the absence of any commercial or financial relationships that could be construed as a potential conflict of interest.

## Footnotes

1 www.diacarbon.com

2 http://github.com/seanmcallister/bin_coverage_tools.git

3 https:github.com/tseemann/barrnap
