# Supplementary material for: DNA-Stable Isotope Probing Shotgun Metagenomics Reveals the Resilience of Active Microbial Communities to Biochar Amendment in Oxisol Soil
Source: Front Microbiol. 2020 Nov 17;11:587972. doi: 10.3389/fmicb.2020.587972 (PMC7717982; doi:10.3389/fmicb.2020.587972)
Supplement: Supplementary file 1 [file Data_Sheet_1.docx]

Supplementary Material

## Supplementary Figures


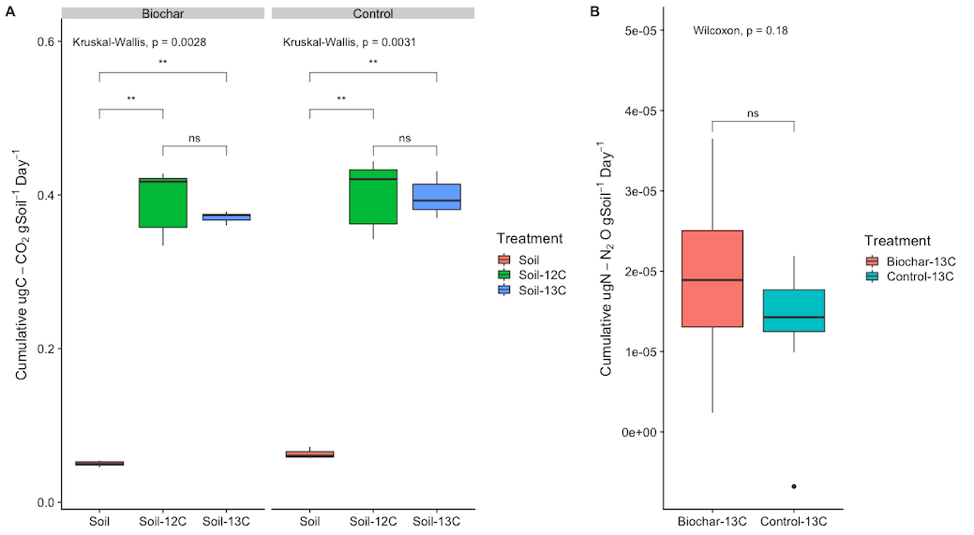


**Supplementary Figure 1.** Cumulative CO_2_ and N_2_O gas for microcosms receiving ^13^C-perennial ryegrass over a 14-day incubation period. Points represent the average microcosm CO_2_ and N_2_O concentration and error bars represent the standard errors of the means (n = 12), inserts show equation for best-fit lines.


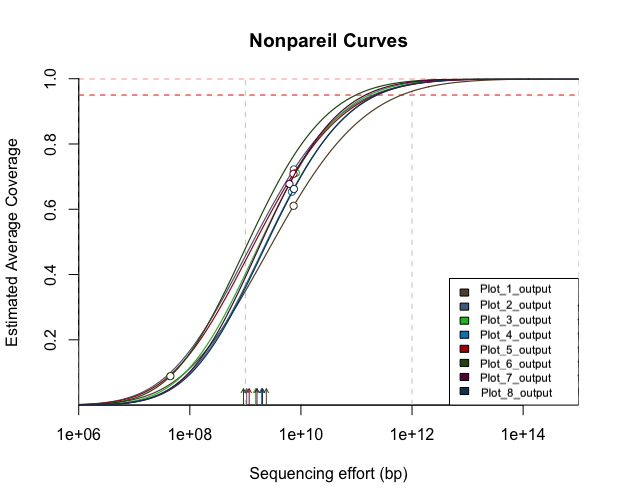


**Supplementary Figure 2.** Average coverage of metagenomes, estimated from the portion of nonunique reads as a function of the size of subsamples randomly drawn from metagenomes of biochar-amended and control soils. Solid lines indicate the fitted models based on subsampling, the open circles mark the actual size and estimated coverage of the metagenomic dataset, red and pink dashed-line indicates the 95% and 100% average coverage levels, respectively.

## Supplementary Tables

**Supplementary Table 1.** Soil properties per plot in microcosms incubated with ^13^C-labeled perennial ryegrass.

|  |  | Biochar-amended | | | | Control | | | |
| --- | --- | --- | --- | --- | --- | --- | --- | --- | --- |
|  |  | Plot 1 | Plot 3 | Plot 4 | Plot 8 | Plot 2 | Plot 5 | Plot 6 | Plot 7 |
| Soil chemicals | Ca (mg/kg) | 1201.2 ± 292.8 | 1651.1 ± 25.6 | 1243.0 ± 47.0 | 2024.5 ± 365.1 | 2070.0 ± 10.0 | 1711.6 ± 4.4 | 1099.0 ± 359.0 | 1432.8 ± 180.8 |
|  | Na (mg/kg) | 32.8 ± 3.9 | 46.1 ± 2.1 | 30.6 ± 2.2 | 37.9 ± 3.3 | 31.1 ± 3.7 | 37.5 ± 9.9 | 30.0 ± 3.4 | 40.8 ± 3.8 |
|  | Mg (mg/kg) | 214.6 ± 26.4 | 285.0 ± 10.1 | 225.9 ± 46.1 | 273.0 ± 8.1 | 185.5 ± 32.5 | 230.2 ± 35.8 | 229.9 ± 70.1 | 236.6 ± 35.4 |
|  | K (mg/kg) | 972.1 ± 17.9 | 671.9 ± 35.3 | 1175.0 ± 107.0 | 923.3 ± 68.7 | 1050.0 ± 164.0 | 789.0 ± 203.0 | 661.0 ± 119.0 | 653.6 ± 49.6 |
|  | C (%)* | 1.84 ± 0.31^ab^ | 2.15 ± 0.45^ab^ | 1.96 ± 0.14^ab^ | 2.39 ± 0.02^a^ | 1.31 ± 0.03^b^ | 1.41± 0.04^b^ | 1.38 ± 0.04^b^ | 1.38 ± 0.02^b^ |
|  | N (%) | 0.17 ± 0. 1 | 0.17 ± 0. 1 | 0.17 ± 0.00 | 0.20 ± 0.01 | 0.16 ± 0.00 | 0.16 ± 0.00 | 0.17 ± 0.00 | 0.17 ± 0.01 |
|  | pH | 6.66 ± 0.54 | 6.99 ± 0.13 | 6.55 ± 0.01 | 6.81 ± 0.20 | 7.40 ± 0.06 | 6.78 ± 0.10 | 5.99 ± 0.52 | 6.46 ± 0.00 |
|  | Moisture (%)^*^ | 43.56 ± 0.66^a^ | 33.55 ± 0.42 ^b^ | 35.40 ± 1.34 ^b^ | 34.17 ± 1.32 ^b^ | 34.33 ± 2.28 ^b^ | 34.90 ± 1.78 ^b^ | 34.08 ± 1.46 ^b^ | 34.08 ± 1.21 ^b^ |

^*^ p<0.05^,^ ** p<0.01^,***^ p<0.001: One way ANOVA, letters indicate Students Newman-Keul post hoc test

**Supplementary Table 2.** Significant and nearly significant results differentially abundant KO terms between biochar-amended and control metagenomes.

| **KO term** | **Base Mean** | **log2Fold**  **Change** | **Lfc SE** | **stat** | **pvalue** | **padj** | **KEGG Family** | **Gene** |
| --- | --- | --- | --- | --- | --- | --- | --- | --- |
| K00370 | 363.29 | -0.432 | 0.105 | -4.128 | 3.66E-05 | 0.03 | 02020 Two-component system [PATH:ko02020] | narG; narZ; nxrA; nitrate reductase / nitrite oxidoreductase; alpha subunit |
| K11891 | 163.55 | 0.528 | 0.123 | 4.275 | 1.91E-05 | 0.03 | 02025 Biofilm formation - Pseudomonas aeruginosa [PATH:ko02025] | impL; vasK; icmF; type VI secretion system protein ImpL |
| K07347 | 127.71 | 0.632 | 0.153 | 4.132 | 3.59E-05 | 0.03 | 05133 Pertussis [PATH:ko05133] | fimD; fimC; mrkC; htrE; cssD; outer membrane usher protein |
| K03286 | 63.82 | 0.602 | 0.155 | 3.882 | 1.04E-04 | 0.06 | 02000 Transporters [BR:ko02000] | TC.OOP; OmpA-OmpF porin; OOP family |
| K11904 | 223.03 | 0.626 | 0.162 | 3.854 | 1.16E-04 | 0.06 | 02044 Secretion system [BR:ko02044] | vgrG; type VI secretion system secreted protein VgrG |
| K06994 | 1887.60 | -0.384 | 0.103 | -3.747 | 1.79 E-04 | 0.07 | 99996 General function prediction only | K06994; putative drug exporter of the RND superfamily |
| K03336 | 326.03 | -0.340 | 0.091 | -3.752 | 1.76 E-04 | 0.07 | 00562 Inositol phosphate metabolism [PATH:ko00562] | iolD; 3D-(3;5/4)-trihydroxycyclohexane-1;2-dione acylhydrolase (decyclizing) |
| K09118 | 369.50 | -0.454 | 0.124 | -3.661 | 2.51 E-04 | 0.08 | 99997 Function unknown | K09118; uncharacterized protein |
| K11896 | 125.59 | 0.466 | 0.128 | 3.635 | 2.78 E-04 | 0.08 | 02044 Secretion system [BR:ko02044] | impG; vasA; type VI secretion system protein ImpG |
| K04768 | 183.95 | -0.432 | 0.122 | -3.551 | 3.83 E-04 | 0.09 | 99981 Carbohydrate metabolism | acuC; acetoin utilization protein AcuC |
| K03466 | 1705.56 | -0.292 | 0.082 | -3.5650902 | 3.64 E-04 | 0.09 | 03036 Chromosome and associated proteins [BR:ko03036] | ftsK; spoIIIE; DNA segregation ATPase FtsK/SpoIIIE; S-DNA-T family |

**Supplementary Table 3.** Characteristics of medium- and high-quality genome bins. Metrics were calculated from CheckM.

| Genome Bin I.D. | Average Bin^a^  Coverage | Taxonomy^b^  (Family-level) | Completeness (%) | Contamination (%) | GC (%) | Size (Mbp) | Coding Density |
| --- | --- | --- | --- | --- | --- | --- | --- |
| Biochar-amended soil | | | | | | | |
| Bin.1_13 | 9.69 | Micrococcaceae | 58.62 | 0 | 67.5 | 2.46 | 91.1 |
| Bin.1_14_1 | 7.52 | Rhizobiaceae | 50.86 | 5.17 | 64.5 | 4.14 | 89.33 |
| Bin.1_17 | 7.23 | Streptomycetaceae | 60.28 | 6.36 | 71.8 | 5.90 | 88.74 |
| Bin.1_18 | 9.34 | Xanthomonadaceae | 70.34 | 3.45 | 70.5 | 2.58 | 91.75 |
| Bin.1_21 | 38.19 | Streptomycetaceae | 71.84 | 5.17 | 73.1 | 8.93 | 88.71 |
| Bin.1_22 | 16.18 | Dermatophilaceae | 89.79 | 3.88 | 71.8 | 3.68 | 91.72 |
| Bin.1_23 | 15.71 | Streptosporangiaceae | 67.83 | 9.05 | 71.6 | 8.72 | 92.46 |
| Bin.1_3 | 8.40 | 20CM-4-69-9 | 84.20 | 3.74 | 70.1 | 3.79 | 93.92 |
| Bin.1_31_1 | 8.50 | Kribbellaceae | 65.16 | 9.48 | 68.9 | 6.79 | 93.17 |
| Bin.1_32 | 21.03 | Catenulisporaceae | 50.63 | 0 | 70.7 | 7.97 | 89.85 |
| Bin.1_33 | 9.15 | 2-12-FULL-66-21 | 75.34 | 2.59 | 68.3 | 4.73 | 91.99 |
| Bin.1_35 | 62.33 | Streptomycetaceae | 61.34 | 3.06 | 71.1 | 4.86 | 90.95 |
| Bin.1_36 | 26.46 | Gemmatimonadaceae | 90.69 | 2.75 | 69.9 | 3.77 | 92.86 |
| Bin.1_37 | 10.67 | Sphingomonadaceae | 55.63 | 5.91 | 64.1 | 1.84 | 92.15 |
| Bin.1_6_1 | 15.22 | Micromonosporaceae | 71.05 | 9.65 | 69.5 | 5.78 | 91.92 |
| Bin.3_15 | 8.74 | Burkholderiaceae | 79.99 | 2.52 | 68.0 | 4.66 | 88.90 |
| Bin.3_16 | 112.83 | Streptomycetaceae | 56.71 | 2.79 | 71.1 | 6.49 | 90.81 |
| Bin.3_19 | 29.67 | Streptomycetaceae | 88.73 | 6.45 | 71.0 | 10.9 | 89.54 |
| Bin.3_21 | 9.38 | Gemmatimonadaceae | 83.15 | 3.85 | 70.2 | 3.68 | 91.43 |
| Bin.3_22_1 | 14.32 | Pseudonocardiaceae | 60.34 | 2.59 | 72.2 | 5.01 | 91.22 |
| Bin.3_25 | 8.30 | Mycobacteriaceae | 77.85 | 1.44 | 68.3 | 5.03 | 89.08 |
| Bin.3_28_1 | 11.83 | Dermatophilaceae | 50.52 | 9.42 | 71.7 | 1.96 | 92.53 |
| Bin.3_29 | 18.84 | Micromonosporaceae | 79.98 | 4.30 | 70.1 | 7.30 | 91.71 |
| Bin.3_37 | 9.01 | Mycobacteriaceae | 66.18 | 1.75 | 68.7 | 4.19 | 90.12 |
| Bin.3_38 | 23.85 | Gemmatimonadaceae | 89.78 | 2.75 | 69.9 | 3.73 | 92.75 |
| Bin.3_6 | 16.81 | Xanthomonadaceae | 58.62 | 6.90 | 67.9 | 2.44 | 93.66 |
| Bin.3_8 | 9.73 | Nocardioidaceae | 63.95 | 0 | 72.9 | 3.64 | 93.21 |
| Bin.3_9 | 14.00 | Rhizobiaceae | 91.14 | 3.42 | 63.1 | 5.06 | 88.75 |
| Bin.4_12_3 | 9.94 | Sphingomonadaceae | 50.34 | 6.03 | 64.0 | 1.94 | 92.31 |
| Bin.4_17_1 | 41.92 | Micromonosporaceae | 82.48 | 6.25 | 70.2 | 6.73 | 92.29 |
| Bin.4_18_1 | 12.46 | Streptosporangiaceae | 56.66 | 9.48 | 71.5 | 10.1 | 93.89 |
| Bin.4_20 | 71.86 | Streptomycetaceae | 67.70 | 2.36 | 70.8 | 8.76 | 90.22 |
| Bin.4_3 | 12.46 | Gemmatimonadaceae | 85.65 | 4.4 | 69.6 | 4.27 | 91.77 |
| Bin.4_31 | 11.82 | Gemmatimonadaceae | 88.09 | 7.74 | 70.3 | 3.54 | 91.90 |
| Bin.4_6_1 | 7.88 | Catenulisporaceae | 63.79 | 8.62 | 70.7 | 8.21 | 90.71 |
| Bin.4_9_1_1 | 9.32 | Xanthobacteraceae | 68.09 | 9.32 | 64.7 | 4.30 | 89.11 |
| Bin.4_30_1_1 | 12.04 | Rhodanobacteraceae | 92.08 | 0.94 | 69.2 | 3.14 | 90.43 |
| Bin.8_14 | 20.20 | Haliangiaceae | 90.45 | 3.39 | 68.3 | 9.81 | 93.72 |
| Bin.8_16 | 17.27 | Polyangiaceae | 94.91 | 5.18 | 66.2 | 11.9 | 91.83 |
| Bin.8_18 | 34.94 | Streptomycetaceae | 64.47 | 0 | 70.5 | 11.3 | 88.38 |
| Bin.8_36 | 34.44 | Micromonosporaceae | 86.21 | 3.28 | 70.3 | 7.13 | 92.28 |
| Bin.8_4 | 16.69 | Xanthomonadaceae | 79.31 | 1.72 | 68.9 | 4.43 | 87.29 |
| Bin.8_40 | 15.16 | Dermatophilaceae | 87.07 | 3.80 | 71.7 | 3.54 | 91.67 |
| Bin.8_42 | 11.74 | Gemmatimonadaceae | 89.85 | 2.75 | 70.2 | 4.10 | 91.37 |
| Bin.8_45 | 78.91 | Streptomycetaceae | 50.86 | 1.72 | 71.1 | 6.84 | 90.94 |
| Bin.8_6 | 31.06 | Gemmatimonadaceae | 90.49 | 2.2 | 69.9 | 3.75 | 92.85 |
| Bin.8_9_1_1 | 8.79 | Polyangiaceae | 73.39 | 8.92 | 67.2 | 8.26 | 94.33 |
| Bin.8_17_1_1 | 8.84 | Rhodanobacteraceae | 73.45 | 6.90 | 69.4 | 3.21 | 90.73 |
| Bin.8_41_1_1 | 46.53 | Streptosporangiaceae | 56.19 | 8.62 | 71.7 | 8.16 | 92.64 |
| Control soil | | | | | | | |
| Bin.2_15 | 9.78 | Streptomycetaceae | 63.98 | 2.90 | 71.9 | 6.57 | 88.73 |
| Bin.2_16 | 9.89 | Microbacteriaceae | 62.56 | 1.35 | 71.5 | 2.11 | 91.89 |
| Bin.2_17 | 7.22 | o_20CM-4-69-9 | 62.41 | 9.48 | 72.2 | 2.37 | 94.19 |
| Bin.2_2 | 48.68 | Pseudonocardiaceae | 58.91 | 2.59 | 71.8 | 9.92 | 91.81 |
| Bin.2_21 | 13.78 | Micromonosporaceae | 77.19 | 7.89 | 71.1 | 7.32 | 90.66 |
| Bin.2_23_2 | 20.43 | Streptosporangiaceae | 69.68 | 6.97 | 71.2 | 7.47 | 93.38 |
| Bin.2_23_3 | 21.83 | Streptosporangiaceae | 59.04 | 0.69 | 71.1 | 4.12 | 93.03 |
| Bin.2_24 | 26.22 | Micromonosporaceae | 96.48 | 1.93 | 69.1 | 7.45 | 91.71 |
| Bin.2_3 | 11.48 | QHCE01 | 95.33 | 1.26 | 58.5 | 3.05 | 90.84 |
| Bin.2_31 | 154.23 | Streptomycetaceae | 71.86 | 0.54 | 70.7 | 8.02 | 89.48 |
| Bin.2_36 | 8.93 | Pseudonocardiaceae | 50.34 | 3.45 | 72.1 | 8.71 | 90.84 |
| Bin.2_7 | 13.36 | Sphingomonadaceae | 88.96 | 8.83 | 64.4 | 2.29 | 93.71 |
| Bin.5_1 | 12.81 | Nocardioidaceae | 90.78 | 5.04 | 72.5 | 4.71 | 92.4 |
| Bin.5_13 | 40.30 | Dermatophilaceae | 74.23 | 0.63 | 71.9 | 3.26 | 91.73 |
| Bin.5_19 | 15.38 | Gemmatimonadaceae | 91.97 | 2.75 | 70.2 | 4.04 | 91.54 |
| Bin.5_20 | 164.13 | Streptomycetaceae | 57.76 | 1.72 | 71.2 | 6.20 | 91.03 |
| Bin.5_27 | 12.34 | Micromonosporaceae | 83.74 | 6.06 | 71.3 | 4.26 | 91.08 |
| Bin.5_30 | 22.91 | Streptosporangiaceae | 76.90 | 9.33 | 71.6 | 8.29 | 92.52 |
| Bin.5_34 | 17.65 | Micrococcaceae | 55.17 | 3.45 | 67.4 | 2.70 | 91.36 |
| Bin.5_5 | 28.65 | Gemmatimonadaceae | 90.69 | 2.75 | 69.9 | 3.77 | 91.91 |
| Bin.6_1_1 | 21.92 | Streptomycetaceae | 85.81 | 9.65 | 70.0 | 11.0 | 87.19 |
| Bin.6_17 | 10.33 | Dermatophilaceae | 55.57 | 1.09 | 71.3 | 2.03 | 92.07 |
| Bin.6_18 | 7.72 | Sphingomonadaceae | 90.50 | 4.27 | 64.9 | 2.34 | 93.07 |
| Bin.6_19 | 8.01 | Micrococcaceae | 72.40 | 2.01 | 68.6 | 2.74 | 91.35 |
| Bin.6_2 | 8.65 | Gemmatimonadaceae | 73.43 | 2.20 | 69.5 | 2.97 | 91.59 |
| Bin.6_3 | 8.27 | Acidobacteriaceae | 90.30 | 1.94 | 58.3 | 5.17 | 89.72 |
| Bin.6_6 | 12.51 | Gemmatimonadaceae | 85.93 | 4.72 | 69.6 | 3.81 | 92.05 |
| Bin.6_9 | 75.61 | Catenulisporaceae | 88.53 | 3.86 | 70.9 | 9.53 | 90.18 |
| Bin.7_11 | 10.13 | Streptomycetaceae | 52.25 | 5.70 | 71.7 | 5.29 | 88.12 |
| Bin.7_12 | 30.29 | Streptosporangiaceae | 81.48 | 7.75 | 71.3 | 9.15 | 92.13 |
| Bin.7_13 | 29.74 | Gemmatimonadaceae | 88.68 | 2.20 | 69.9 | 3.71 | 92.85 |
| Bin.7_14 | 8.84 | Mycobacteriaceae | 76.00 | 1.42 | 68.3 | 5.18 | 89.09 |
| Bin.7_19_2 | 20.78 | Streptomycetaceae | 50.86 | 8.19 | 71.2 | 10.9 | 89.14 |
| Bin.7_20 | 23.97 | Dermatophilaceae | 89.76 | 5.89 | 71.7 | 3.72 | 91.77 |
| Bin.7_21 | 12.05 | Burkholderiaceae | 87.47 | 0.31 | 68.0 | 4.83 | 88.69 |

^a^ Bin coverage is calculated using perl script. Bin coverage is weighted by the length

^b^ Taxonomy determined using GTDB-Tk at the family level
